# Risk prediction models to guide antibiotic prescribing: a study on adult patients with uncomplicated upper respiratory tract infections in an emergency department

**DOI:** 10.1186/s13756-020-00825-3

**Published:** 2020-11-02

**Authors:** Joshua Guoxian Wong, Aung-Hein Aung, Weixiang Lian, David Chien Lye, Chee-Kheong Ooi, Angela Chow

**Affiliations:** 1 Department of Clinical Epidemiology, Office of Clinical Epidemiology, Analytics and Knowledge, Tan Tock Seng Hospital, Singapore, Singapore; 2 Infectious Disease Research and Training Office, National Centre for Infectious Diseases, Singapore, Singapore; 3 Department of Infectious Diseases, Tan Tock Seng Hospital, Singapore, Singapore; 4 Lee Kong Chian School of Medicine, Nanyang Technological University, Singapore, Singapore; 5 Yong Loo Lin School of Medicine, National University of Singapore, Singapore, Singapore; 6 Department of Emergency Medicine, Tan Tock Seng Hospital, Singapore, Singapore; 7 Saw Swee Hock School of Public Health, National University of Singapore, Singapore, Singapore

**Keywords:** Adult, ED, URTI, Antibiotic prescribing, Machine learning, Prediction model

## Abstract

**Background:**

Appropriate antibiotic prescribing is key to combating antimicrobial resistance. Upper respiratory tract infections (URTIs) are common reasons for emergency department (ED) visits and antibiotic use. Differentiating between bacterial and viral infections is not straightforward. We aim to provide an evidence-based clinical decision support tool for antibiotic prescribing using prediction models developed from local data.

**Methods:**

Seven hundred-fifteen patients with uncomplicated URTI were recruited and analysed from Singapore’s busiest ED, Tan Tock Seng Hospital, from June 2016 to November 2018. Confirmatory tests were performed using the multiplex polymerase chain reaction (PCR) test for respiratory viruses and point-of-care test for C-reactive protein. Demographic, clinical and laboratory data were extracted from the hospital electronic medical records. Seventy percent of the data was used for training and the remaining 30% was used for validation. Decision trees, LASSO and logistic regression models were built to predict when antibiotics were not needed.

**Results:**

The median age of the cohort was 36 years old, with 61.2% being male. Temperature and pulse rate were significant factors in all 3 models. The area under the receiver operating curve (AUC) on the validation set for the models were similar. (LASSO: 0.70 [95% CI: 0.62–0.77], logistic regression: 0.72 [95% CI: 0.65–0.79], decision tree: 0.67 [95% CI: 0.59–0.74]). Combining the results from all models, 58.3% of study participants would not need antibiotics.

**Conclusion:**

The models can be easily deployed as a decision support tool to guide antibiotic prescribing in busy EDs.

**Supplementary Information:**

The online version contains supplementary material available at 10.1186/s13756-020-00825-3.

## Background

Upper respiratory tract infection (URTI) is one of the most cited reasons for use of antibiotics [1]. In the majority of URTIs, the routine use of antibiotics is not recommended [1–5]. In the United States (U.S.), it was estimated that antibiotics have been prescribed for over 60% of uncomplicated URTIs in adults and increasingly so for broad-spectrum antibiotics [6–9]. Between 2001 and 2010, 126 million (12.2%) emergency department (ED) visits in the U.S. were for acute respiratory tract infections, with almost half (47.9%) of patients with infections being administered antibiotics inappropriately [10]. From 2009 to 2010, adults had the highest rate of inappropriate antibiotic use for acute respiratory tract infections (URTIs, influenza, and viral pneumonia), with 500 antibiotic prescriptions per 1000 ED visits for adults aged 20–64 years and 666 per 1000 visits for those aged > = 65 years [10].

In Singapore, while primary care clinics are highly accessible in the community, there are individuals who preferred to seek care at the ED for URTI, accounting for a substantial proportion of ED attendances [11]. URTI accounted for 6–10% of ED visits by non-frequent attenders (1–4 ED visits in one year) and up to 25% of ED visits by frequent attenders (≥5 ED visits in one year) [12]. A previous study at an adult general hospital has reported that 24% of adult patients attending at ED for URTI were inappropriately prescribed antibiotics, with the penicillin class of antibiotics being the most commonly prescribed [13].

Studies have shown a strong link between antibiotic prescribing and antimicrobial resistance [6, 14, 15]. In addition, a population-wide study on US pharmacy records showed that antibiotic use and resistance appears to be closely linked to broadly distributed low-intensity prescribing [16]. As a consequence, antimicrobial resistance has risen to dangerously high levels globally. A global study estimated that *Escherichia.coli* and *Klebsiella pneumoniae* resistant to third-generation cephalosporin caused 6.4 million bloodstream infections and 50.1 million serious infections in 2014. Carbapenem-resistant strains were estimated to cause 0.5 million bloodstream infections and 3.1 million serious infections [17]. Antimicrobial resistance is associated with higher medical cost, prolonged hospital stays, increased mortality and economic burden [18, 19]. Hence, there is an urgent need to ensure the prudent use of antibiotics for common illnesses predominantly of viral etiology such as URTIs.

In Singapore, considerable efforts have been made to address antibiotic resistance [20]. Although computerized decision support systems have been developed to guide antibiotic prescribing, they are largely based on guidelines drawn by expert consensus and not on actual data derived from local patients [21]. Furthermore, most studies on antibiotic prescribing focus on understanding behaviors and perceptions or finding associative factors for antibiotic prescribing decisions [22–25]. To date, prediction models to guide antibiotic prescribing has been confined largely to pediatric populations [26–28].

Differentiating bacterial and viral infections is not straightforward in adult URTIs. In uncertainty avoidance, physicians tend to over-prescribe antibiotics. In this study, we aim to develop prediction models based on local clinical and laboratory data to guide antibiotic prescribing for adult patients with uncomplicated URTI with the ultimate goal of deploying them as an evidence-based clinical decision support tool for routine practice.

## Methods

### Patient cohort

Seven hundred-fifteen patients were recruited from the ED at Tan Tock Seng Hospital (TTSH), the second largest adult hospital in Singapore between June 2016 and November 2018. Eligible patients were 21 years and above attending at TTSH ED for the first time with a primary diagnosis of uncomplicated URTI (ICD10-AM J00-J06) within 30 days who provided informed consent. TTSH ED is the busiest ED in the country, attending to an average of 450 patients daily.

### Selection of participants

At discharge from the emergency department, the patients were invited to participate in the study and consent was obtained. Patients who were subsequently admitted were excluded from the study. A nasopharyngeal swab was taken to determine the presence of respiratory viruses using multiplex PCR (Seeplex® RV15 ACE Detection). The panel detects 15 major respiratory viruses including adenovirus, bocavirus 1/2/3/4, coronavirus 229E/NL63 and OC43, enterovirus, influenza A and B, metapneumovirus, parainfluenza 1, 2, 3 and 4, respiratory syncytial virus A and B, and rhinovirus. We chose not to include the bacterial respiratory PCR panel in the study, as commensal bacteria are common in the upper respiratory tract and detection on PCR does not necessarily indicate a bacterial infection. A lower respiratory tract sample (such as sputum) was also not practicable for every participant. Instead, we performed a point-of-care C-reactive protein (CRP) test on a drop of capillary blood obtained from a finger prick (QuikRead go® CRP). CRP is widely used in clinical settings as a supportive test to diagnose bacterial infection [29].

### Outcomes

Our main outcome of interest was to identify patients for whom antibiotics were clearly not recommended (1 = NABX) from those for whom the physician should review the need for antibiotics (0 = RABX). We defined NABX as patients with a respiratory virus detected via PCR **and** CRP < 20 mg/L or patients who did not have a respiratory virus detected via PCR **and** CRP ≤ 5 mg/L [30]. Patients who did not fall into these 2 categories were assigned to the RABX group.

### Dependent variables

Demographic, clinical and laboratory data documented as part of the patients’ routine care were extracted from the hospital electronic medical records. These include age, gender, ethnicity, visit date, pre-existing comorbidities, respiratory symptoms, full blood count, kidney/liver panels, and biochemistry tests. According to comorbid status of the participants, Charlson’s Comorbidity Index was calculated [31]. Additionally, epidemiologic data on smoking, influenza vaccination, travel history, and prior medical consultation and antibiotic consumption were obtained from an interviewer-administered questionnaire.

### Statistical analysis

Descriptive statistics were performed and differences between the NABX and RABX groups compared using Mann-Whitney U-test for continuous variables and Chi-squared test for categorical variables. Where appropriate, Fisher’s exact tests were used to account for small cell sizes. Variables with more than 10% of data missing were excluded from the analysis. Categorical variables with data missing were recoded as 0 under the assumption that presence of any clinical covariates would have been recorded. Continuous variables were imputed according to their group medians.

With ease of use in mind, we decided to perform predictive modeling using 3 methods that could subsequently be easily deployed for implementation: logistic regression, LASSO regression and classification and regression trees (CART). The models were derived using 70% of the participants as training set. The optimal cutoffs for each model were decided by taking the predicted probability that achieved the highest sensitivity with specificity of at least 0.4. The final model performance was validated by calculating the area under the receiver operating characteristic curve (AUC), sensitivity, specificity, positive predictive value and negative predicative values on the remaining 30% data.

#### Logistic regression

Univariate analysis was performed on all 50 candidate variables. Demographic factors, clinically relevant variables and significant variables from univariate models were fitted into the final multivariable model via stepwise elimination using a cutoff of *p* < 0.1.

#### Lasso

In stepwise regression, it is often difficult to tell the effect after removal of each variable. Model selection may also be difficult in datasets with a huge number of variables. LASSO regression addresses this by shrinking the coefficients of features that are less relevant or exhibit collinearity to zero. This reduces the problem of overfitting of prediction model and the variance without substantial increase in bias. We performed this by selecting a minimum optimal shrinking parameter of λ = 0.03971531 through a 10-fold cross validation of the training dataset, giving a set of coefficients governed by Eq. 1.

$$ {\hat{\beta}}_{\lambda }=\underset{\beta }{\mathit{\arg}\mathit{\min }}\left(-\sum \left[{y}_i{x}_i\beta -\mathit{\log}\left(1+\mathit{\exp}\left({x}_i\beta \right)\right)\right]+\lambda \sum \left|{\beta}_j\right|\right),\lambda >0 $$

#### CART

CART is a popular tool in supervised learning for classification as they are distribution-free and robust to outliers. Unlike generalized linear models, classification trees make an excellent tool for overcoming problems due to multicollinearity and skewed covariates. It uses the Gini index to iteratively split branches based on purity. This feature is an added benefit as important interactions can be easily detected. It also has the ability to identify patient subgroups that are more predictive than others. In our analysis, we created a maximum tree depth of 5 and a minimum of 10 subjects in a node before a split is attempted to prevent overfitting. The choice of the final tree size was decided by finding the number of splits that produce the smallest cross-validation error.

Analyses were performed using R4.0.2 and STATA 13.0 at a 5% significance level. LASSO and CART models were developed using the *glmnet*, *rpart* and *rattle* packages in R [32–34].

## Results

### Characteristics of study subjects

**Fig. 1:**
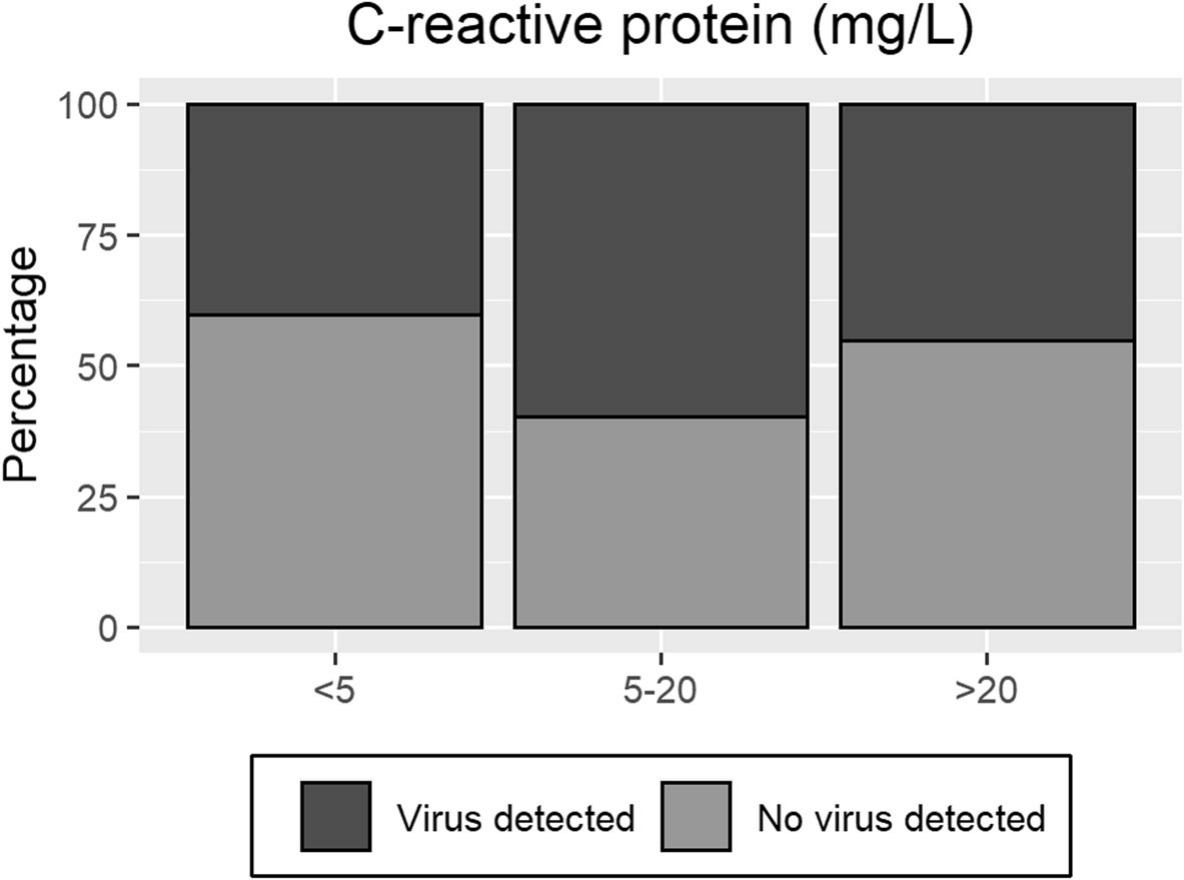
Summary of virus positivity by C-reactive protein levels

The study participants were young, with a median age of 36 years (IQR: 28–51 years) and a slight preponderance of males (61.3%). (Table 1) Two-thirds (66.4%) had no pre-existing comorbidities and one-third (36.8%) had received influenza vaccination in the prior 12 months. Almost two-thirds (60%) of the patients presented with fever. While 50.3% of the patients had nasal problems like running and blocked nose, 45.6% of them had a sore throat. Almost half (47.8%) of the patients had a respiratory virus detected. Influenza (20.6%) and rhinovirus (14.4%) were common respiratory viruses detected. Influenza circulated year-round, with bimodal peaks observed in November and May–June, with rhinovirus dominating in the inter-influenza periods. (data not shown) The median CRP level was 6 mg/L (IQR 4–19 mg/L) and its natural logarithm had a correlation of 0.022 with the viral status. Patients with a detectable result on the PCR had higher CRP levels at 8 mg/L (IQR: 4–19 mg/L) as compared to those without at 4 mg/L (IQR: 4–19 mg/L) (*p* = 0.041). Among patients with low (< 5 mg/L), moderate (5–20 mg/L) and high (> 20 mg/L) levels of CRP, approximately 40, 60 and 45% of them respectively had a detectable result on PCR (Fig. 1). In total, 461 (64.5%) patients were classified as NABX (Fig. 1).

**Table 1:** Clinical Signs and Symptoms of patients diagnosed with URTI and recommendations on need for antibiotics. The actual counts (percentage) and median (IQR) are reflected for categorical and continuous variables respectively

Variables	Review of antibiotics recommended (RABX) (*n* = 254)	Antibiotics not recommended (NABX) (*n* = 461)	*p*
**Demographics**			
Age (years)	37 (29–54)	36 (28–50)	0.170
Gender			0.009
Male	172 (67.7)	266 (57.7)	
Residency			0.815
Residents	185 (72.8)	332 (72.0)	
Race			0.110
Chinese	111 (43.7)	197 (42.7)	
Malay	40 (15.7)	98 (21.3)	
Indian	56 (22.0)	74 (16.0)	
Others	47 (18.6)	92 (20.0)	
Visit Month			0.211
January	11 (4.3)	35 (7.6)	
February	15 (5.9)	24 (5.2)	
March	19 (7.5)	31 (6.7)	
April	24 (9.4)	27 (5.9)	
May	20 (7.9)	38 (8.2)	
June	14 (5.5)	42 (9.1)	
July	29 (11.4)	69 (15.0)	
August	24 (9.4)	40 (8.7)	
September	34 (13.4)	45 (9.8)	
October	31 (12.2)	51 (11.1)	
November	14 (5.5)	34 (7.4)	
December	19 (7.5)	25 (5.4)	
**Epidemiologic Data**			
Smoker	53 (20.9)	114 (24.7)	0.123
Influenza Vaccination in the past 1 year	94 (37.0)	169 (36.7)	0.675
Travelled Overseas	74 (29.1)	116 (25.2)	0.250
Prior Consultation in 14 days	146 (57.5)	228 (49.5)	0.040
Prescribed antibiotics during prior consultation	64 (25.2)	125 (27.1)	0.578
**Comorbidities**			
Asthma	36 (14.2)	91 (19.7)	0.062
COPD	40 (15.7)	77 (16.7)	0.741
Taking steroids	12 (4.7)	44 (9.5)	0.032
Diabetes mellitus	25 (9.8)	39 (8.5)	0.535
Liver disease	3 (1.2)	9 (2.0)	0.554
Cancer	11 (4.3)	8 (1.7)	0.039
Myocardial Infarction	8 (3.1)	12 (2.6)	0.671
Chronic Heart Failure	2 (0.8)	2 (0.4)	0.618
Renal disease	4 (1.6)	5 (1.1)	0.728
Charlson’s Comorbidity Index >0	82 (32.3)	158 (34.3)	0.590
**Symptoms**			
Onset of symptoms (days)	4 (3–7)	4 (3–7)	0.438
Fever	188 (74.0)	234 (50.8)	< 0.001
Headache	27 (10.6)	38 (8.2)	0.288
Joint pain	8 (3.1)	9 (2.0)	0.315
Abdomen pain	14 (5.5)	16 (3.5)	0.193
Loss of appetite	23 (9.1)	35 (7.6)	0.493
Body ache	48 (18.9)	52 (11.3)	0.005
Diarrhea	10 (3.9)	18 (3.9)	0.983
Runny nose	116 (45.7)	244 (52.9)	0.063
Nausea	12 (4.7)	26 (5.6)	0.602
Red eye	3 (1.2)	4 (0.9)	0.704
Rash	7 (2.8)	11 (2.4)	0.763
Shortness of breath	38 (15.0)	107 (23.2)	0.009
Sore throat	130 (51.2)	196 (42.5)	0.026
Vomiting	21 (8.3)	19 (4.1)	0.021
Giddiness	8 (3.1)	33 (7.2)	0.027
Tiredness	13 (5.1)	17 (3.7)	0.361
**Signs**			
Conjunctival congestion	3 (1.2)	6 (1.3)	1.000
Dehydration	15 (5.9)	16 (3.5)	0.126
Injected pharynx	80 (31.5)	111 (24.1)	0.032
Sinus congestion	3 (1.2)	2 (0.4)	0.353
Enlarged tonsil	6 (2.4)	9 (2.0)	0.714
Abnormal findings on abdominal examination	8 (3.1)	7 (1.5)	0.145
Abnormal findings on lungs examination	19 (7.5)	50 (10.8)	0.145
**Vital Signs**			
Highest body temp (°C)	37.4 (36.9–38.4)	37.0 (36.6–37.3)	< 0.001
Highest pulse rate (beats per minute)	96 (84–101)	89 (80–98)	< 0.001
Highest respiratory rate (breaths per minute)	18 (17–18)	18 (17–18)	0.322
Lowest Sa02 level (%)	98 (96–99)	98 (97–99)	0.001
Lowest systolic blood pressure (mmHg)	117 (105–131)	122 (111–133)	< 0.001
Lowest diastolic blood pressure (mmHg)	65 (58–75)	68 (62–77)	0.010

### Univariate analysis

Baseline covariates were largely similar between patients in the RABX and NABX groups. Patients were less likely to have prior consultation 14 days before the ED visit in the NABX group compared with the RABX group (49.5% vs 57.5%, *p* = 0.04). Influenza vaccination uptake rates were similar in both groups (37.0% vs 36.7%, *p* =   0.675). There was no evidence of comorbidity being associated with antibiotic need, except those with steroid use and cancer. Median time from earliest symptom onset to ED visit was similar between both groups at 4 days (IQR: 3–7 days). RABX patients were more likely to present with symptoms of fever, body ache, sore throat and vomiting. NABX patients were likely to display symptoms of shortness of breath and giddiness. Patients in the RABX group had a higher median maximum body temperature and lower median systolic and diastolic blood pressures than those in the NABX group (Table 1).

### Prediction models

**Fig. 2:**
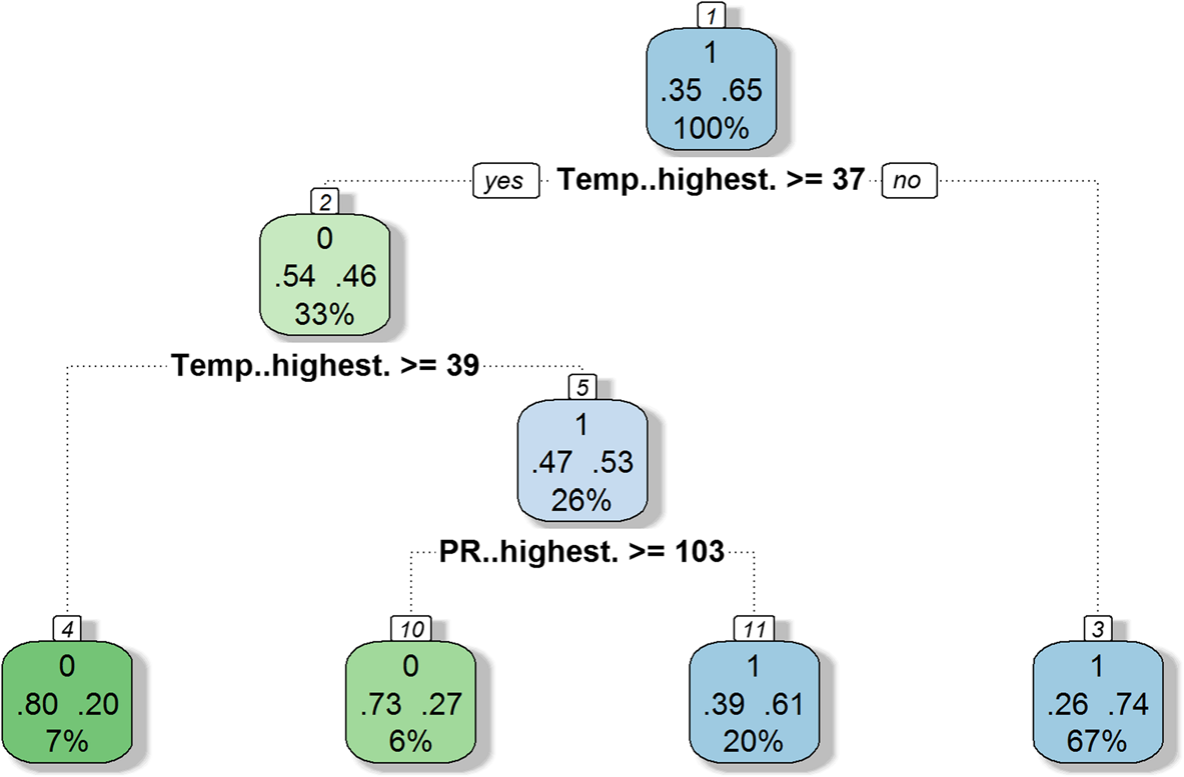
Algorithm for decision tree

**Fig. 3:**
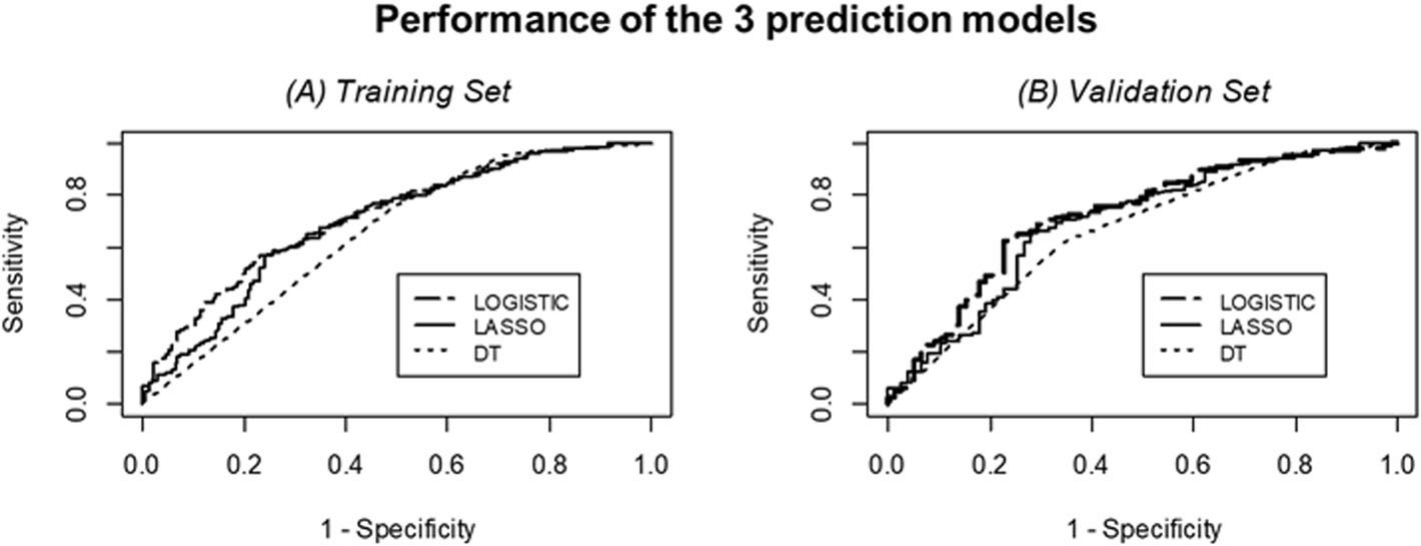
ROC curves for the 3 models

Highest temperature and highest pulse rate were commonly identified to be important predictors in all logistic, LASSO and CART models (Table 2; Fig. 2). In addition, age, the presenting symptoms of fever, giddiness and shortness of breath were identified to be significant predictors in the final logistic regression model. Similarly, Indian ethnicity, fever, giddiness and cancer status were included in the LASSO model. (Table 2) The AUC on the validation set for all three models varied slightly with the highest value of 0.72 (95% CI: 0.65–0.79) for the logistic model, followed by 0.70 (95% CI: 0.62–0.77) for the LASSO model, then 0.67 (95% CI: 0.59–0.74) for the CART model. (Fig. 3) Using a predicted probability cutoff of 0.6, 0.625, and 0.675 for the logistic, LASSO, and CART models respectively, the logistic model produced a sensitivity (Sen) of 0.72 (95% CI: 0.64–0.79) and a specificity (Spe) of 0.65 (95% CI: 0.53–0.75) on the validation set. In contrast, the corresponding values in the LASSO model were 0.72 (95% CI: 0.64–0.79) and 0.62 (95% CI: 0.50–0.73) respectively. Among the three models, the CART model had the highest sensitivity of 0.77 (95% CI: 0.68–0.84) but was the least specific compared with the other 2 models at 0.49 (95% CI: 0.39–0.59). The positive predictive value (PPV) and negative predictive value (NPV) were respectively the smallest and largest for the CART (PPV = 0.62; NPV = 0.66) compared to the logistic and LASSO models (logistic: PPV = 0.78, NPV = 0.56; LASSO: PPV = 0.77, NPV = 0.55). (Table 3).

**Table 2:** Coefficients from the LASSO and logistic regression models

Variables	LASSO	Logistic regression
Age	NA	−0.019 (−0.033 to −0.005)
Indian	−0.088	NA
History of Cancer	−0.182	NA
Giddiness	0.009	1.343 (0.178 to 2.507)
Fever	−0.248	−0.607 (−1.056 to −0.158)
Shortness of Breath	NA	0.551 (0.010 to 1.092)
Highest Temperature	−0.423	−0.456 (−0.755 to −0.157)
Highest Pulse rate	−0.011	−0.029 (− 0.046 to − 0.012)

**Table 3:** Performance of the 3 models at the optimal cutoff

Model	Training set				Validation set			
	Sen	Spe	PPV	NPV	Sen	Spe	PPV	NPV
Logistic	0.78	0.51	0.74	0.56	0.72	0.65	0.78	0.56
LASSO	0.78	0.52	0.75	0.56	0.72	0.62	0.77	0.55
CART	0.76	0.50	0.74	0.54	0.77	0.49	0.62	0.66

In addition, we looked at the corresponding metrics at a probability cut-off of 0.5. The models have marked improvement in sensitivity, but specificity fell below 0.5 (Logistic: Sen = 0.88, Spe = 0.34; LASSO: Sen = 0.94, Spe = 0.27; CART: Sen = 0.95, Spe = 0.29). Detailed documentation on different probability cutoffs can be found in Additional File 1.

## Conclusion

A qualitative study previously conducted in our hospital revealed that ED physicians were confident with their clinical decisions. However, doctors had a lower threshold for prescribing antibiotics for older patients who were immunocompromised and suffering from chronic conditions. Junior physicians were observed to be uncomfortable not prescribing antibiotics for URTI patients [22]. Patients with bacterial and viral infections present with similar symptoms and differentiation of patients requiring antibiotics from those who do not is problematic. Our algorithms developed using three rigorous statistical methods together with laboratory-based confirmatory tests served as a good guide for physicians in their decisions on antibiotic prescribing for URTI patients. A recent Cochrane Systematic Review provided evidence that patient satisfaction and clinical outcomes were similar between those for whom antibiotic prescribing was delayed and those not prescribed antibiotics at all. Delayed prescribing of antibiotics has been found to be associated with marked reduction in antibiotic use [35]. Our results showed that the performance of all 3 prediction models were similarly modest. While we tried to be pragmatic with our algorithms, we also carried out similar analysis on more complex classification trees and random forests, both of which showed minimal or no improvement in performance (AUC ~ 0.7). A recent systematic review showed that there was minimal improvement using machine learning techniques over traditional regression models [36].

Relevant literature on prediction models for antibiotic prescribing in adults are limited and tended to focus on life-threatening infections. Several clinical prediction models were built for pneumonia and serious bacterial infections in children mostly using either logistic regression or decision trees [27, 37, 38]. The findings from this study add to the limited knowledge on clinical decision support tools for antibiotic prescribing in an adult ED setting.

We found that fever and pulse rate were significant factors in all 3 models. Most studies on viral respiratory infections have focused on influenza with a high temperature identified as a significant risk factor in both younger and older adults [39–42]. Heart rate was found to be significant in a group of patients presenting with influenza-like illness at a hospital emergency department [43]. A significant proportion of patients with influenza infection present with tachycardia. This could be due to the physiologic response to fever although cardiac manifestations are not uncommon with complications of influenza [44]. Shortness of breath and giddiness were also found to be significant predictors in two of our models. While these symptoms could be non-specific, we believe that it would have to be significant enough for adult patients to volunteer these symptoms to their physicians when they had them. As physicians often have to make antibiotic prescribing decisions based on subjective symptoms reported by their patients, we believe that our clinical decision support tool, developed from three different models, will provide physicians with a reliable tool when making antibiotic prescribing decisions for patients with URTI at the point-of-care.

There are a few limitations in our study. Firstly, the ability to predict well is dependent on the richness of the data. Our study is limited to the information obtained at the time that the patient medically attended at ED. Knowledge on baseline vital signs and trajectories prior to ED visit may be important information that could improve our models. A 2017 study by Stanford University on wearable devices detected that anomalies in skin temperature and heart rate corresponded to periods of high CRP levels [45]. Secondly, we did not consider laboratory parameters like full blood count, renal and liver function panels in our model as 48% of patients did not receive a full blood count, and even fewer had renal and liver function tests. To address this, we created new variables simulating if full blood count or renal function panel were performed, postulating that doctors may have ordered these tests on patients who warranted further investigation. However, we could not detect any statistical differences. Nevertheless, our models represented real-life situations where such tests were not commonly ordered for uncomplicated URTI patients and the results would often not be factored into the antibiotic decision making by physicians. Thirdly, our study was based at a tertiary hospital and need to be validated in other settings to ensure generalizability. Patients might seek care in the ED after medical attendances at primary care clinics failed to alleviate their symptoms. Our data reflected this as 50% of patients had prior consultation although the time between the earliest symptom onset to ED visit was only 4 days on average. The local literature on vaccination uptake in the community is limited. To our knowledge, there is only one population health survey on influenza vaccination uptake in older adults done in 2013 [46]. The authors found that the influenza vaccination uptake in this population was only 15.2%. Our patient cohort had a higher vaccination rate (37%) than in the community. However, this does not invalidate our findings and we believe that the impact on the generalizability of our models is minimal.

Nonetheless, our study had its strengths. We were able to take seasonality into account as the study spanned two years covering two influenza seasons each of Northern and Southern Hemispheres. The use of PCR together with appropriate CRP cutoffs were based on findings from several international studies and selected to be the most conservative estimates. The cutoff point for CRP was set lower to increase sensitivity of the RABX group [30, 47, 48]. We also note that the proportion of positive viral PCR among the CRP < 5 and CRP > 20 groups were quite similar (Fig. 1). Patients with high CRP and positive viral PCR represent patients with secondary bacterial infection. In a sub-group analysis of 229 patients with complete blood count performed, those with high CRP levels of > 20 and positive viral PCR were almost twice as likely to have leukocyte counts of > 9.6 × 10^9^/L as those with CRP < 20 and positive viral PCR (39.3% vs. 20.2%, *p* = 0.003). This supports our exclusion of patients with high CRP and positive viral PCR from the NABX group. Comprehensive assessment of medical records was performed by two clinically trained individuals with standardization in data extraction methods and definitions to ensure data accuracy and consistency. Analysing the data with 3 different methods not only allowed us to compare models but also allowed us to triangulate the findings from all our models. Notably, maximum pulse rate and highest temperature were considered as important variables in all 3 models. Finally, our models were either coefficient or rule based. They can easily be entered into an excel sheet or the hospital electronic system without the need to integrate complicated programming codes.

**Fig. 4:**
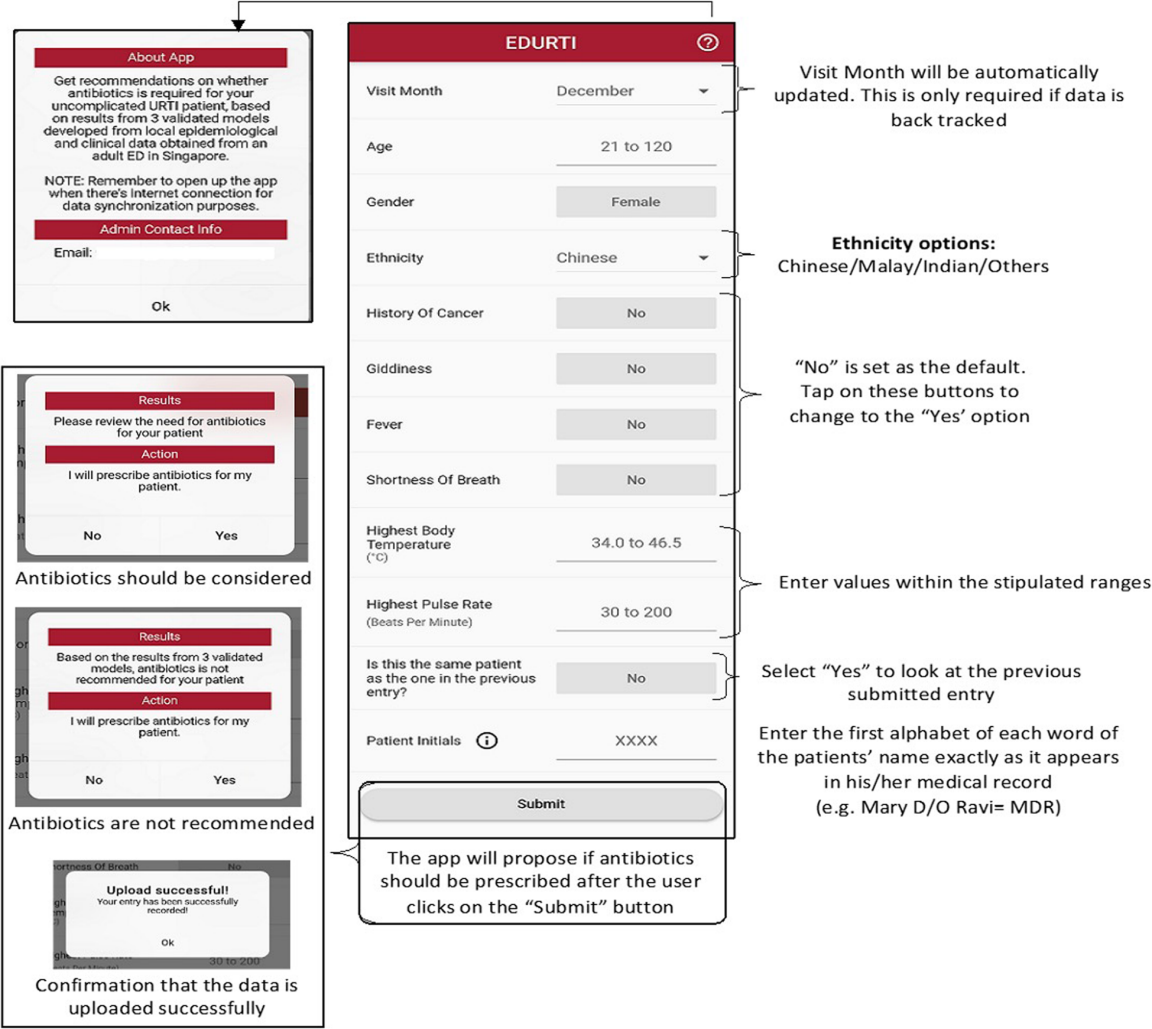
User interface for the “Abx SteWARdS” app

Combining the results from the three models, 58.3% of study participants would not need antibiotics. Moving forward, physicians could use this tool as a useful complement to their clinical judgement in their practice to guide their decisions on antibiotic prescribing. Antibiotics should be prescribed with caution even during low influenza periods as there are still other viruses circulating throughout the year. At the time of writing, we have developed a mobile application (app) named the “Abx SteWARdS” to provide clinical decision support for busy physicians practicing in the ED on antibiotic prescribing for URTI (Fig. 4). ED physicians are required to fill in 9 parameters all on one screen. All fields are mandatory, and the app will provide a recommendation either to review the need for antibiotics or that antibiotics was not needed, based on the predicted outcomes of all 3 validated models. A validation study is underway. It is hoped that evidence-based clinical decision support tools accessible at the point-of-care can lead to better antibiotic prescribing decisions and the reduction of antibiotic resistance.

## Supplementary Information

**Additional file 1: Appendix 1A.** Training diagnostic performance at different probability cutoffs. **Appendix 1B.** 2 x 2 tables for the actual vs predicted values on the training and validation set using the best probability cutoff

## Data Availability

All data generated or analysed during this study are included in this published article (and its Additional file 1).
