# Supplementary material for: Risk prediction models to guide antibiotic prescribing: a study on adult patients with uncomplicated upper respiratory tract infections in an emergency department
Source: Antimicrob Resist Infect Control. 2020 Nov 2;9:171. doi: 10.1186/s13756-020-00825-3 (PMC7605344; doi:10.1186/s13756-020-00825-3)
Supplement: Supplementary file 1 — Additional file 1: Appendix 1A. Training diagnostic performance at different probability cutoffs. Appendix 1B. 2 x 2 tables for the actual vs predicted values on the training and validation set using the best probability cutoff [file 13756_2020_825_MOESM1_ESM.docx]

**Appendix 1A: Training diagnostic performance at different probability cutoffs**

| Predictive Model | Probability Cut-off | Diagnostic | Training Set |
| --- | --- | --- | --- |
| Logistic | 0.5 | Sensitivity | 0.88 |
|  |  | Specificity | 0.34 |
|  |  | PPV | 0.71 |
|  |  | NPV | 0.61 |
|  | 0.6 | Sensitivity | 0.78 |
|  |  | Specificity | 0.51 |
|  |  | PPV | 0.74 |
|  |  | NPV | 0.56 |
|  | 0.625 | Sensitivity | 0.75 |
|  |  | Specificity | 0.54 |
|  |  | PPV | 0.75 |
|  |  | NPV | 0.54 |
| LASSO | 0.5 | Sensitivity | 0.94 |
|  |  | Specificity | 0.27 |
|  |  | PPV | 0.70 |
|  |  | NPV | 0.71 |
|  | 0.625 | Sensitivity | 0.78 |
|  |  | Specificity | 0.52 |
|  |  | PPV | 0.75 |
|  |  | NPV | 0.56 |
|  | 0.65 | Sensitivity | 0.70 |
|  |  | Specificity | 0.62 |
|  |  | PPV | 0.77 |
|  |  | NPV | 0.53 |
| CART | 0.5 | Sensitivity | 0.95 |
|  |  | Specificity | 0.29 |
|  |  | PPV | 0.71 |
|  |  | NPV | 0.77 |
|  | 0.675 | Sensitivity | 0.76 |
|  |  | Specificity | 0.50 |
|  |  | PPV | 0.74 |
|  |  | NPV | 0.54 |

**Appendix 1B: 2 x 2 tables for the actual vs predicted values on the training and validation set using the best probability cutoff**

| **Model** | **Probability Cut-off** | **Predicted** | **Actual** | | | |
| --- | --- | --- | --- | --- | --- | --- |
|  |  |  | **Training** | | **Validation** | |
|  |  |  | RABX | NABX | RABX | NABX |
| Logistic | 0.6 | RABX | 89 | 71 | 51 | 40 |
|  |  | NABX | 86 | 248 | 28 | 102 |
| LASSO | 0.625 | RABX | 91 | 71 | 49 | 40 |
|  |  | NABX | 84 | 248 | 30 | 102 |
| CART | 0.675 | RABX | 88 | 87 | 52 | 27 |
|  |  | NABX | 75 | 244 | 54 | 88 |
